# Preliminary study on fluid bolus administration for prevention of spinal hypotension in dogs undergoing elective cesarean section

**DOI:** 10.3389/fvets.2023.1112845

**Published:** 2023-03-21

**Authors:** Agnieszka Antończyk, Zdzisław Kiełbowicz, Wojciech Niżański, Małgorzata Ochota

**Affiliations:** ^1^Department and Clinic of Surgery, Faculty of Veterinary Medicine, Wrocław University of Environmental and Life Sciences, Wrocław, Poland; ^2^Department of Reproduction and Clinic of Farm Animals, Faculty of Veterinary Medicine, Wrocław University of Environmental and Life Sciences, Wrocław, Poland

**Keywords:** cesarean section, dogs (*Canis familiaris*), epidural analgesia, hypotension, crystalloids coload

## Abstract

**Introduction:**

This study aimed to investigate the effect of fluid bolus administration during epidural anesthesia (coload) in female dogs scheduled for elective cesarean section (CS). Hypotension is one of the most common complications of epidural (EA) and spinal (SA) analgesia, and in the case of cesarean section, it may pose a significant risk for placental perfusion and subsequent fetal vitality and puppy survival.

**Methods:**

Pregnant bitches scheduled for elective CS underwent EA with (treatment group) or without (control group) intravenous fluid bolus administration. The following parameters were measured and compared between both groups: HR, RR, etCO_2_, SpO_2_, systolic, diastolic and mean arterial blood pressure were measured at three time points (T1: before surgery, T2: after the last puppy removal, and T3: end of surgery) in dams; vitality (Apgar score at 0, 5, and 20 min) and umbilical cord blood parameters (pH, pCO2, HCO3, base excess, lactate and glucose) in newborns.

**Results:**

The results indicated that crystalloid coloading increased maternal systolic, diastolic, and mean blood pressure (treatment vs. control group, 101.46 ± 9.18, 48.01 ± 13.47, and 67.07 ± 13.15 mmHg vs. 80.68 ± 7.29, 36.52 ± 8.75, and 180 52.30 ± 7.77, *p* < 0.05) with significantly fewer episodes of hypotension. Additionally, puppies in the treatment group received higher scores in the 5-min (7.91 ± 1.67 vs. 6.74 ± 2.20) and 20-min (9.38 ± 0.87 vs. 8.39 ± 2.50) assessments without the favorable effect on umbilical blood gas parameters.

**Discussion:**

Based on the obtained results, it can be stated that crystalloid coload offers an effective option in cases of hypotension during cesarean section, with clear benefits for both mothers and newborns.

## 1. Introduction

Hypotension is one of the most common complication of epidural (EA) and spinal (SA) analgesia resulting from arterial vasodilation rather than decreased venous return and cardiac output, leading to decreased vascular resistance (1–4).

It has been reported that during cesarean section (CS), hypotension following EA can occur in up to 80% of women (2–4); similar findings have also been noted in dogs (5–10). The consequences of low blood pressure affect both, the mother and unborn fetus. In the first place, epidural hypotension may be associated with nausea and the risk of vomiting in the mother. Additionally, rare cases can cause cardiovascular collapse, loss of consciousness, or aspiration of gastric content (if not intubated) (11). Furthermore, prolonged maternal hypotension can have deleterious effects on uteroplacental perfusion and subsequent acute fetal distress resulting in fetal hypoxia, bradycardia, and acidosis (12–16).

Previous studies have proposed multiple strategies for preventing hypotension after EA in pregnant women (17–33). Various combinations of fluid types, infusion rates, and timing were considered and tested with variable outcomes. Hence, optimal preventive strategies against hypotension during CS, are yet to be determined. Traditionally, crystalloid fluids have been administered before EA for CS (preload) (33, 34). However, some evidences suggested that the rapid redistribution of crystalloids from the vascular bed to the interstitial space makes this method not completely satisfactory (17–20, 29, 35). Therefore, a different approach was attempted with the administration of crystalloids at the point when the local block was about to start (coload). This approach improved the filling of the vasculature at the time of maximum vasodilation caused by the epidural or spinal blockade, and was therefore more effective in preventing hypotension (17–20). On the other hand, some authors noted that patients with colloid preload had fewer episodes of low blood pressure than those patients with colloid coloads (21, 22). In other studies, both groups were similarly hypotensive (23–27). In summary, in human medicine there is still no consensus on the type of fluid and its loading time in patients undergoing EA or regarding the total fluid and dosing rate in preventing epidural hypotension.

Interestingly, despite the similar risk of epidural-induced hypotension in veterinary and human patients, treatment strategies for dogs have received little attention until date. Bosman et al. studied colloid (HES 6%) loading in healthy, non-pregnant dogs (28). Fluids were administered at a constant infusion rate for 30 min prior to epidural administration of ropivacaine. Unfortunately, this strategy did not mitigate hypotension episodes in dogs. The main limitation of the present study was the small number of animals investigated therefore, future studies involving a larger study population and administration other types of fluids, different doses, and administration rates are needed.

During CS procedures, the risk of hypotension is high, and can be caused by anesthetic, sedative, and analgesic drugs, as well as the patient's dorsal recumbency during surgery, traction on ovarian pedicles, or sudden removal of the uterus from the abdomen. The combined effects of these factors can lead to severe hypotension. To minimize this risk, one of the recommended strategies is to avoid epidural-induced hypotension (16, 36).

Since there are no references to colloid/crystalloid preload or coload dosing in dogs and there is a wide variation in doses administered to humans, the authors decided to evaluate the effectiveness of crystalloid coload in preventing intraoperative hypotension in dogs undergoing CS and evaluate whether fluid administration would have a beneficial effect on newborn puppy outcomes evaluated based on the Apgar scoring system and umbilical cord blood gas results.

## 2. Material and methods

### 2.1. Animals

The study protocol was approved by the II Local Ethics Committee for Animal Experiments (No 047/2020). The twenty four client-owned bitches of different breeds scheduled for elective cesarean section were enrolled in this study. Bitches with a previous history of ongoing health concerns unrelated to pregnancy or developing health problems during pregnancy were excluded from the study. The cardiovascular parameters were checked on admission: heart rate, thorough auscultation of heart sounds and rhythm, capillary refill time, and mucous membrane color. No abnormalities were found. The dogs were healthy, and without any systemic diseases based on routine clinical, hematological, and biochemical examinations. The mean body weight of the females was 14.6 kg (3–46 kg), and the mean age was 4.1 (2–8) years. Cesarean section was performed independently in this research. Clinical indications (e.g., single-pup pregnancy), previous history of dystocia, or breed related factors (brachycephalics) were considered when scheduling the patient for CS. Elective cesarean section was performed on day 64 (±1) of gestation, according to standard guidelines including determination of LH peak, presence of milk, fetal heart rate, and its gastrointestinal motility.

### 2.2. Anesthetic and experimental protocol

The dams were randomly assigned to one of two groups: C, control group (without fluid load) and T, treatment group (with fluid load). All females underwent the same standard anesthetic protocol. The bitches received a single dose of meloxicam (0.2 mg/kg s.c., Metacam 5 mg/ml, Boehringer Ingelheim, Poland) at least 30 min prior to the surgical procedure. The dams were preoxygenated for 3–5 min, and without premedication, general anesthesia was induced with propofol (Propofol-Lipuro^®^, 10 mg/ml B. Braun Melsungen AG, Germany, initial dose 4 mg/kg, to effect) and the dams were intubated. General anesthesia was maintained with isoflurane (IsoVet^®^, Piramal Healthcare, United Kingdom) in oxygen. Subsequently, the dogs were positioned in sternal recumbency with the pelvic limbs extended cranially. After aseptic preparation of the skin, a spinal needle was introduced into the lumbosacral space and 3 mg/kg lidocaine was administered (Lignocainum Hydrochloricum WZF 2%, Polfa Warszawa, Poland). Correct placement of the needle was confirmed by the presence of a distinct ‘popping sensation’ as a result of penetrating the ligamentum flavum, the lack of resistance to injection, and the absence of cerebrospinal fluid and blood in the needle hub. Intravenous fluids (crystalloids) were infused at a rate of 5 mL/kg/h during anesthesia in all dogs. An IV bolus of fluid (crystalloid or colloid at 5–10 or 3–5 mL/kg, respectively) was administered when hypotension occurred. Furthermore, in the treatment group (group T), the dams received 15 mL/kg of Ringer Lactate Solution (Fresenius Kabi, Warszawa, Poland), starting at the time of epidural blockage (coload) and administered within 15 min. Throughout the infusion and surgical procedure, females were carefully monitored for signs of fluid overload (chemosis, serous nasal discharge, reduced breath sounds, and/or coarse crackles).

After transfer to the surgical theater and complete instrumentation, monitoring was started and the following parameters were continuously monitored and noted at 2 min intervals: heart rate (HR), respiratory rate (RR), end-tidal CO_2_ (etCO_2_), oxygen saturation (SpO_2_), systolic, diastolic and mean arterial blood pressure (SBP, DBP, MBP, respectively), minimal alveolar concentration of isoflurane (MAC) and esophageal body temperature (T), (Datex-Ohmeda S5 monitor Helsinki, Finland).

To compare hemodynamic and respiratory changes over time, three time points were considered: T1, immediately before the skin incision, T2, after the last puppy removal, and T3, at the end of surgery. The values were obtained as the mean of 3-4 consecutive measurements observed in each animal during time points T1, T2, and T3.

The surgical procedure was carried out with a standard midline approach. After the was removed uterus from the abdominal cavity, the incision was made on the ventral or dorsal surface of the uterine body. After removal of the puppies', the uterus was sutured with a single continuous pattern (Monosyn^®^ 3/0, B. Braun Aesculap Chifa Sp. z o.o., Nowy Tomyśl, Poland). The abdominal cavity was flushed with warm saline before closing. The abdominal wall, subcutaneous tissue, and skin were routinely closed using a synthetic absorbable material.

### 2.3. Umbilical cord sampling for blood gas analysis

Umbilical cord blood sampling was performed as previously described (5, 6). Directly after the puppy extraction from the uterus, the fetal membranes were torn and removed from the muzzle and the umbilical cord was double clamped before placenta detachment. At least 100 mcl of mixed umbilical cord blood was collected in a heparinized syringe with a 25G needle directed away from the puppy and immediately analyzed using EPOC VET handheld analyzer (Siemens Healthineers, Germany), a point-of-care diagnostic device certified for veterinary use.

### 2.4. Apgar scoring

The Apgar evaluation was performed t by the same experienced staff member. We used a modified Apgar scoring system adapted for bitches. The first scoring was performed immediately after delivery, before any neonatal assistance was instituted (0 min), and thereafter at 5 and 20 min. Finally, each newborn underwent a full clinical evaluation prior to discharge (~90 min after the end of the surgery). The following reference ranges for AS were used for Apgar score: HR >220 bpm (2 points), 180–220 bpm (1 point) and <180 (0 points); RR >15 breaths per minute (2 points), 6–15 (1 point) and <6 (0 points); irritability reflex detected after gentle compression of the tip of a paw was evaluated based on the degree of reaction: crying and quick leg retraction (2 points), weak leg retraction and no or just weak vocalization (1 point), and no leg retraction and vocalization (0 points); spontaneous movement of a newborn: strong movement (2 points), weak movement (1 point), and absent (0 point); mucous membrane color: dark pink (2 points), light pink (1 point) and pale to cyanotic (0 point). The total points received provided the final AS: 7–10, no distress, healthy newborns; 4–6, moderate distress, weak newborn and 0–3, severe distress, critical newborns (37).

### 2.5. Statistics

Statistical analysis included descriptive statistics and normality testing using the Kolmogorov–Smirnov test with Lilliefors correction. Non-parametric tests were performed when the results did not follow a normal distribution. Pre- and intraoperative parameters of the dams and umbilical blood gas parameters were compared the between groups using *t*-test. Intra-group differences between considered time points were measured using analysis of variance (ANOVA) for dependent variables. Apgar scores were compared between the groups using the U Mann–Whitney test.

## 3. Results

The mothers in both groups did not differ in terms of mean age, body weight, preoperative respiratory rate (RR), body temperature, dose of propofol required for the induction of general anesthesia, and number of pups per litter (Table 1). The baseline mean value of maternal heart rate (HR) for the two groups was comparable statistically. Furthermore, there was no statistically significant difference (*p* = 0.55) in the total anesthetic time (from the induction to the end of the surgery) between the control (53 ± 15.12 min) and treatment (55.22 ± 12.08 min) groups. Similarly, isoflurane demand was comparable in all investigated females (MAC 1.13 ± 0.19 vs. 1.22 ± 0.14, *p* = 0.41). No signs of fluid overload were observed in any of the mothers who received fluid coload.

**Table 1:** General clinical data and preoperative parameters in bitches without (control group) or with (treatment group) fluid coload.

**Dams—general data**		**Control group**	**Treatment group**	***p*-value**
Parameter		Mean (range)		
Age (years)		3.7 (2–8)	4.4 (2–8)	0.38
Body weight (kg)		12.4 (3–32)	16.9 (2.5–46)	0.34
Pups per litter		3.9 (1–9)	4.3 (1–12)	0.37
Preoperative	HR (bpm)	114 (80–168)	126 (84–160)	0.24
	RR (breaths/min)	55.6 (24–120)	72.7 (22–120)	0.21
	T (°C)	38 (37.2–38.5)	37.8 (36.7–38.5)	0.55
Propofol dose (mg/kg)		5.9 (3.4–8.3)	4.7 (1.3–6.6)	0.13

HR, heart rate; RR, respiratory rate; T, body temperature measured in the esophagus.

Intraoperative parameters such as HR, RR, etCO2 and body temperature were also similar in all females. In contrast, blood pressure differed between the groups. In the control group, the mean values of SBP, DBP and MBP were 80.68 ± 7.29, 36.52 ± 8.75, and 52.30 ± 7.77 mmHg, respectively. Up to 91.6% of the investigated females developed hypotension, defined as a mean arterial pressure below 60 mmHg. In dams who received fluid coload, blood pressure was higher (101.46 ± 9.18, 48.01 ± 13.47 and 67.07 ± 13.15 mmHg for SBP, DBP, and MBP, respectively, *p* < 0.05) with significantly fewer episodes of hypotension (33%, *n* = 4).

**Figure 1:**
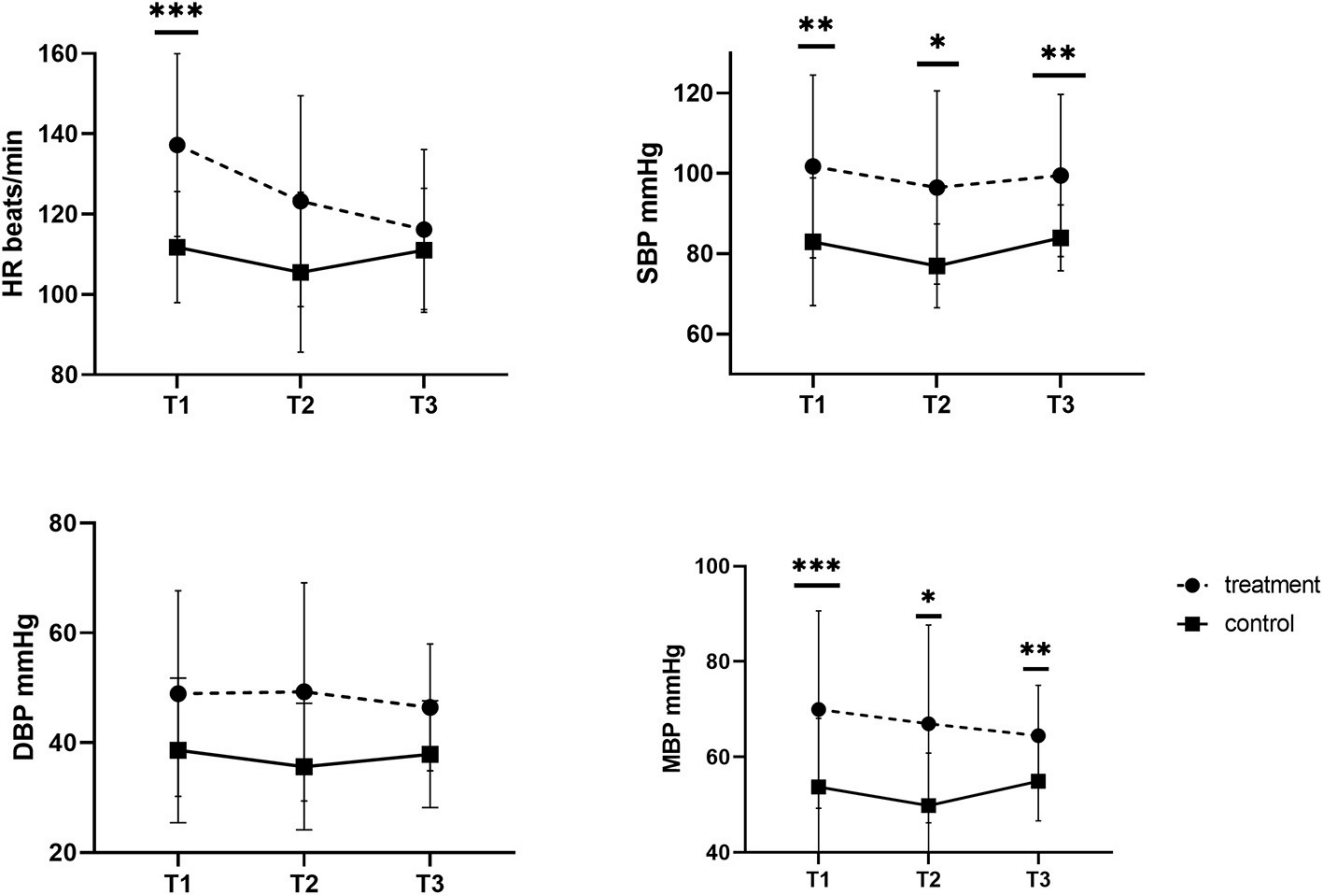
Systolic, diastolic, and mean blood pressure (SBP, DBP, MBP) and heart rate (HR) in dams without (control group) and with (treatment group) fluid coload at different time points. *p = 0.01. **p = 0.02. ***p = 0.03.

Figure 1 shows the mean values of SBP, DBP and MBP tracings observed in the control and treatment groups during anesthesia for cesarean section. In both groups, SBP was the lowest after the removal of all puppies (T2), but in the treatment group blood pressure was significantly higher at this time point (T: 96.50 ± 24.05 vs. C: 76.98 ± 10.42 mmHg). A similar significant difference was noted for T1 and T3. The DBP did not change over time in all investigated females (*p* > 0.05), or between the investigated groups at all considered time points. In the treatment group, MBP dropped slightly from T1 (70.00 ± 20.67 mmHg) to T3 (64.47 ± 10.57 mmHg), nevertheless it was still higher than that in the control group at each of the investigated time points.

**Figure 2:**
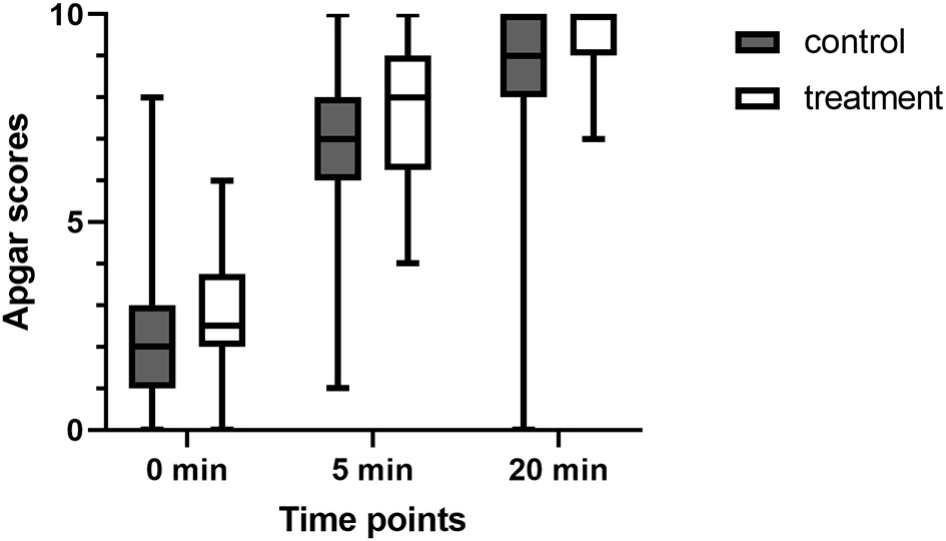
Comparison of Apgar scores (AS) at 0, 5, and 20 min after birth in puppies from dams who received (treatment group) or not (control group) fluid coload.

The number of puppies included in the study was 42 in the control group and 33 in the treatment group. Stillborn puppies and those with failed umbilical cord blood collection were excluded from the experiment. No differences were found in any umbilical blood gas parameters in the newborns of either group (Table 2). However, the neonatal viability assessment differed significantly, and the puppies in group T received higher scores in the 5-and 20-min assessments (Figure 2).

**Table 2:** The umbilical blood gas values of the puppies from dams who received (treatment group) or not (control group) fluid coload.

	**pH (units)**	**pCO_2_ (mmHg)**	**HCO^3−^(mmol/L)**	**BE (b) (mmol/L)**	**Lac (mmol/L)**	**Glu (mg/dl)**
Control	7.195 ± 0.07	59.39 ± 14.01	22.64 ± 3.90	−5.96 ± 4.40	2.67 ± 1.32	75.59 ± 28.47
Treatment	7.185 ± 0.07	64.22 ± 11.56	24.06 ± 2.08	−5.49 ± 3.09	2.70 ± 1.25	74.03 ± 15.58
*p*-value	0.59	0.11	0.08	0.6	0.92	0.77

pCO_2_, partial pressure of carbon dioxide; HCO^3−^, concentration of bicarbonate; BE, base excess; Lac, concentration of lacate; Glu, concentration of glucose.

## 4. Discussion

The present study, for the first time, showed the efficacy of crystalloid fluids coloading in dogs undergoing CS in preventing epidural-induced hypotension. Our results showed, that both SBP and MBP were significantly higher in the treatment group that received coload fluid treatment than those in the control group without additional fluid administration. Furthermore, episodes of hypotension were significantly less frequent in dams that received a fluid bolus (33 vs. 91.6%). Similar improvement in blood pressure have been widely reported in human medicine (17–20, 29). However, in the present study, similar to human reports, it was not possible to completely prevent episodes of maternal hypotension (17–20, 29). Nevertheless, we noted that the treated bitches, which received crystalloid coload at the same time as EA, had an overall higher blood pressure, and their newborns vitality scores at 5 and 20 min were also significantly better compared to the control group without fluid coload administration. Furthermore, in the treatment group, we observed an increase in HR at the first post-infusion point (T1). This observation can be explained by the Bainbridge reflex. According to this theory, the subsequent increase in venous return after rapid fluid infusion causes activation of the right atrial stretch receptors, triggering an increase in the HR (38). Such an effect might be beneficial for fetal circulation and welfare, and thus the subsequent newborn survival, since placental and fetal perfusion depends solely on maternal blood pressure (39).

We found that the investigated parameters of the umbilical cord blood gas analysis were similar in both the treatment and control groups. However, a comprehensive comparison of the obtained results poses some difficulty. Available data on the umbilical cord blood gas evaluation in dogs are limited. To the best of our knowledge, no reports have been published on the effect of maternal fluid infusion during anesthesia on newborn puppies' condition. Only human studies have reported no pH changes in umbilical cord blood after crystalloid or colloid preload administration to the mother before spinal anesthesia (32, 33, 40). In the present study, to assess neonatal outcomes, we measured not only umbilical cord pH, but also base excess and lactate concentration, as important markers confirmed in human medicine that indicate peripartum fetal stress. In the results presented, neither base excess nor lactate concentration showed differences between the investigated and control groups. Furthermore, pCO2, HCO3, and glucose levels were also similar in the T and C groups, and were comparable to those reported previously in dogs (5, 6). These data suggest that crystalloids infusion in dams at a rate of 15 mL/kg during EA might be ineffective for the improvement of umbilical blood gas in their newborn puppies.

Similarly, the blood gas parameters obtained indicated no differences in puppies' vitality immediately after delivery (AS at 0 min 2.2 ± 1.9 in the control vs. 2.6 ± 1.5 in the treatment group, *p* > 0.05). However, in the following evaluations of newborn vitality at 5 and 20 min, significant differences were found in AS between the treatment and control groups. The newborns of the treatment group had a higher AS both at 5 and 20 min than the puppies born from the dams of the control group (AS at 5 min: 6.7 ± 2.2 in the control vs. 7.9 ± 1.8 in the treatment group, *p* = 0.02; AS at 10 min: 8.3 ± 2.5 vs. 9.3 ± 0.9, *p* = 0.01). A possible explanation for this could be the results described by Hollmen et al. for humans (41). Babies in the group of lower maternal blood pressure after epidural analgesia had no or weak reflexes up to the 2nd day of life, compared to newborns of normotensive mothers, despite the similar initial ASs recorded in both groups. However, in most human investigations no differences in AS were observed (32, 33, 40) regardless of maternal blood pressure. Unfortunately, to date, no such study has been conducted in dogs. Similar to Hollmen et al. we found that, the differences in newborns' postnatal improvement between hypo- and normotensive dams were not apparent at the first post-delivery vitality assessment but they became evident over time. As explained by Hollmen et al. even short-term hypotension leads to decreased placental blood flow that persists for several minutes after the correction of maternal hypotension. Such a situation can cause neurological depression and abnormal postpartum neurological recovery.

The results presented here showed that crystalloids infusion, administered together with EA (coload), increased maternal blood pressure. Crystalloid coload was also beneficial for the vitality of the puppies and increased ASs at 5 and 20 min without a favorable effect on blood gas parameters. However, it was not possible to prevent maternal hypotension completely. Therefore, despite the administration of fluid bolus, maternal blood pressure should be carefully monitored throughout surgery and clinicians must be prepared for rescue hypotension treatment during CS.

## Data availability statement

The original contributions presented in the study are included in the article/supplementary material, further inquiries can be directed to the corresponding author.

## Ethics statement

The animal study was reviewed and approved by Bioethics Committee of the Hirszfeld Institute of Immunology and Experimental Therapy. Written informed consent for participation was not obtained from the owners because this study included client-owned bitches scheduled for elective cesarean section. The cesarean section was decided independently of this research, solely on clinical indications (e.g., single pup pregnancy), the previous dystocia in history, or breed-related factors (brachycephalics).

## Author contributions

AA: conceptualization, data collection, data evaluation, statistical analysis, and writing of the manuscript. MO: conceptualization, data collection, data evaluation, and reviewing and editing the paper. WN and ZK: supervising the study and reviewing and editing the paper. All authors contributed to the article and approved the submitted version.
